# Combined Application of Leguminous Green Manure and Straw Determined Grain Yield and Nutrient Use Efficiency in Wheat–Maize–Sunflower Rotations System in Northwest China

**DOI:** 10.3390/plants13101358

**Published:** 2024-05-14

**Authors:** Na Zhao, Lanfang Bai, Dongxun Han, Zhiyuan Yao, Xiaodong Liu, Yaru Hao, Zhipeng Chen, Xiaohong Zhang, Dongrui Zhang, Xiaoling Jin, Zhigang Wang

**Affiliations:** 1 College of Agronomy, Inner Mongolia Agricultural University, Hohhot 010019, China; 2 Bayannur Academy of Agricultural & Animal Sciences, Linhe 015400, China; 3 Key Laboratory of Mountain Surface Processes and Ecological Regulation, Institute of Mountain Hazards and Environment, Chinese Academy of Sciences, Chengdu 610041, China; 4 College of Public Administration, Inner Mongolia University, Hohhot 010021, China

**Keywords:** leguminous green manure, straw return, fertilization management, crop yield

## Abstract

Leguminous green manure (LGM) has a reputation for improving crop productivity. However, little is known about the beneficial interactions with straw on crop yield and nutrient (N, P, K) use efficiency. Herein, a 9-year field experiment (from 2015 to 2023) containing three treatments—(1) chemical fertilizer as the control (CK), (2) NPK + straw return (Straw) and (3) NPK + straw return with LGM (Straw + LGM)—was conducted to investigate whether the combined application of LGM and straw can increase productivity and nutrient use efficiency in the wheat–maize–sunflower diversified cropping rotation. The results showed that in the third rotation (2021–2023), Straw + LGM significantly increased wheat yield by 10.2% and maize yield by 19.9% compared to CK. The total equivalent yield under Straw + LGM was the highest (26.09 Mg ha^−1^), exceeding Straw and CK treatments by 2.7% and 12.3%, respectively. For each 2 Mg ha^−1^ increase in straw returned to the field, sunflower yield increased by 0.2 Mg ha^−1^, whereas for each 1 Mg ha^−1^ increase in LGM yield from the previous crop, sunflower yield increased by 0.45 Mg ha^−1^. Compared to CK, the co-application of LGM and straw increased the N use efficiency of maize in the first and third rotation cycle by 70.6% and 55.8%, respectively, and the P use efficiency by 147.8% in the third rotation cycle. Moreover, Straw treatment led to an increase of net income from wheat and sunflower by 14.5% and 44.6%, while Straw + LGM increased the net income from maize by 15.8% in the third rotation cycle. Combining leguminous green manure with a diversified cropping rotation has greater potential to improve nutrient use efficiency, crop productivity and net income, which can be recommended as a sustainable agronomic practice in the Hetao District, Northwest China.

## 1. Introduction

The rapid increase in chemical fertilizer use, together with its overuse or misuse, with low nutrient use efficiency in about a third of the global cropland area, has caused widespread environmental pollution and economic loss worldwide [1,2]. In China, farmers apply 550–600 kg N ha^−1^ annually for maize and wheat, significantly surpassing the combined N demand of both crops, which is about 330 kg N ha^−1^ [3]. Also, phosphorus (P) fertilizer application has increased by five times from 1980 to 2014, but the augmentation in overall grain yield was merely twofold, with a low P fertilizer yield effect [4,5]. P use efficiency for food crops is only 11.8%, with 30.0% for potassium (K) use efficiency in China [6]. Nutrient management has emerged as a primary focus of agronomy, in relation to crop yield, efficiency, the environment and the economics of modern farming [2]. Nutrient use efficiency in plants determines whether they can efficiently uptake nutrients from the soil [7]. Improving nutrient use efficiency—nutrient input harvested as product—is one of the most effective means of increasing crop productivity while decreasing environmental pollution [8]. Numerous studies have reported that improving nutrient utilization efficiency is closely related to agricultural management [9,10,11].

Crop rotation and intercropping are common management approaches currently being explored to reinforce the sustainability and yield of agriculture [12,13,14]. In contrast to monocultures or double-farmed rotations, diversified crop rotations refer to a set of or multiple rotations of three or more crops [9]. Carefully selecting a crop rotation scheme has the potential to reduce trade-offs between crop viability and environmental impacts, maintain long-term soil fertility, and disrupt the weed and disease cycle process through intrinsic nutrient recycling [12]. The rotation effect has been demonstrated regardless of whether the crop rotation comprises legumes or non-leguminous plants. For example, intercropping, known as a low environmental cost production strategy [13], is recommended to be widely practiced in temperate regions and is considered a fundamental principle for the sustainable intensification of agriculture, particularly under the current scenario of climate change [15]. Likewise, it has been well documented that the yields of maize and wheat are lower in a monoculture than in a crop rotation [16].

The application of straw to agricultural land has been viewed as a good way to recycle nutrients and organic matter that can support crop production [17]. Nevertheless, research in South Asia found that due to the slow rate of crop residue decay [18] and to a lack of synchrony of N release with crop demand during crop growth [19], grain yield and nutrient use efficiency decreased with wheat straw incorporation when compared with straw removal [19]. Intercropping leguminous green manure (LGM), such as hairy vetch, is a productive planting system that can make full use of environmental resources and respond to the low nutrient use efficiency of farmland soil in contemporary agricultural production [20]. The positive effect of legumes on crop yield has been confirmed through a large number of field experiments [10,21] and is primarily due to the improvement of N recovery from the LGM. For example, legumes can fix atmospheric N_2_ [22]. Long-term experiments showed that a year-round application of legumes in the rotational system resulted in higher N use efficiency (up to 37%) compared to fallow land [23]. However, another study suggested that LGM negatively affects crop growth, yield and harvest in sequential crops [24]. LGM also slows down the overapplication of chemical fertilizers and N leaching and alleviates soil salinity [25]. A study conducted in Hetao Irrigation District revealed that a wheat–green manure multiple cropping system can effectively enhance soil organic carbon, thereby mitigating soil salinity [25]. Nevertheless, there is a paucity of comprehensive research on the effects of LGM on the balance between crop yield and nutrient use efficiency, as well as the economic benefits, when LGM is incorporated into cropping systems (e.g., intercropping or crop rotation), particularly in saline–alkali soil [26].

Sustainable intensification of cropping systems is essential to achieve global food security and environmental security [27], and, based on local natural resources, development of new cropping systems with matching agronomic management (fertilizer input, tillage, etc.) can maintain high yields while reducing environmental costs [28]. Nevertheless, finding efficient combinations of agroecosystem traits for a given pedoclimatic and socioeconomic context remains difficult. For instance, the incorporation of crop residues as straw in rotations can lead to N losses as high as synthetic fertilizers [14]. The Hetao Irrigation District is one of the important grain production bases in China, with an annual sowing area of about 9.0 × 10^4^ ha of wheat and a total output of more than 4.5 × 10^8^ kg. In addition, it has an annual sowing area of about 3.0 × 10^5^ ha of sunflower and a total output of about 8.0 × 10^8^ kg [4]. The Hetao Irrigation District plays an important role in guaranteeing the food security of China, but also faces multiple challenges [26,29]. Development of intensive agricultural systems has been associated with the overuse of large quantities of chemical fertilizers and growth of the same crop for long periods of time in the pursuit of economic efficiency. Crop monoculture has detrimental effects on soil health and crop yield, posing significant challenges in maintaining agronomic productivity and soil nutrient levels [25,30] (e.g., increased soil salinization). Severe soil salinization may lead to a reduction in crop productivity in the Hetao Irrigation District [31]. Sustainable cropping systems (such as diversified crop rotations) with nutrient management practices to ensure food security and develop resilient cropping systems are needed urgently to enhance local agricultural development.

Therefore, the objective of this study was to comprehensively evaluate the effects of combined LGM/straw and chemical fertilizer (chemical fertilizer only, chemical fertilizer and straw, chemical fertilizer and straw combined with LGM) applications on nutrient use efficiency, crop yield and economic income of spring wheat, maize and sunflower in a three-crop rotation experiment.

## 2. Results

### 2.1. Yield and Yield Components

**Figure 1:**
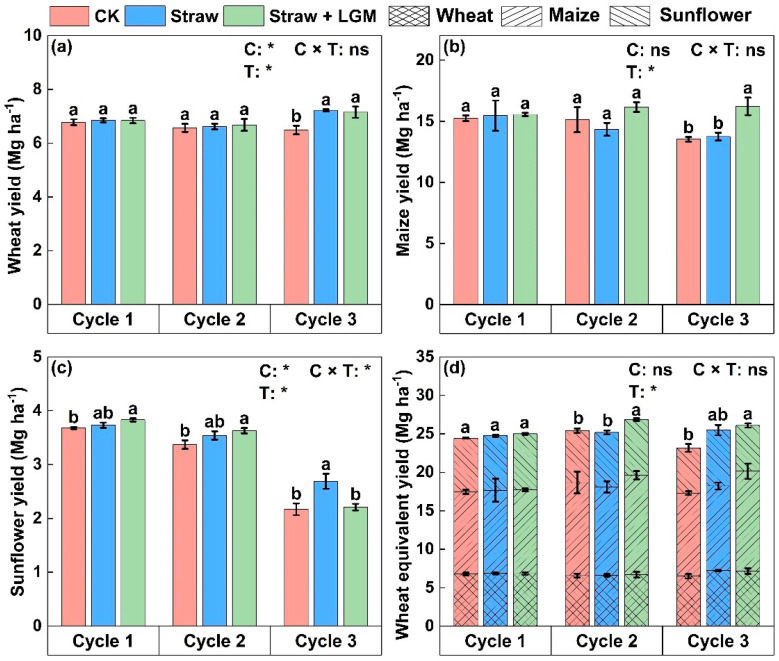
The yields of wheat (**a**), maize (**b**) and sunflower (**c**), as well as the wheat equivalent yield of the spring wheat–maize–sunflower rotation system (**d**) with straw and with leguminous green manure (LGM) in three rotation cycles. The different lowercase letters indicate significant differences between the fertilization treatments at the *p* = 0.05 level. CK: chemical fertilizer alone; Straw: chemical fertilizer with returned straw; Straw + LGM: chemical fertilizer with returned straw and leguminous green manure; C: rotation cycle; T: treatment; * means *p* < 0.05; ns means *p* > 0.05.

The application of chemical fertilizer and combined application of straw and LGM significantly influenced the grain yield of wheat, maize and sunflower (Figure 1, *p* < 0.05). Specifically, there was no significant difference in wheat yield in the first and second rotation cycles between Straw and Straw + LGM treatments. In the third rotation cycle, wheat yield under Straw and Straw + LGM (7.22 and 7.15 Mg ha^−1^, respectively) was significantly higher than that for the CK (6.49 Mg ha^−1^) (Table 1). The maize yield under Straw + LGM (16.22 Mg ha^−1^) was significantly greater than that for the Straw and CK (13.53–13.75 Mg ha^−1^) in the third cropping rotation cycle (*p* < 0.05). In the first and second rotation cycles, the sunflower yield under Straw + LGM (3.83 and 3.63 Mg ha^−1^, respectively) was significantly greater than that under Straw (3.73 and 3.53 Mg ha^−1^, respectively) and CK (3.68 and 3.37 Mg ha^−1^, respectively). In the third rotation cycle, the sunflower yield under Straw (2.69 Mg ha^−1^) was greater than Straw + LGM (2.21 Mg ha^−1^) and CK (2.17 Mg ha^−1^), with yield increases of 0.7 times. In the second rotation cycle, spike number of wheat was significantly higher under Straw + LGM (719.1 m^−2^) than Straw (694.2 m^−2^; Table 2, *p* < 0.05). In the third rotation cycle, the highest grain number was recorded under Straw + LGM (41.2 spike^−1^), followed by Straw (38.7 spike^−1^) and CK (35.4 spike^−1^). Hundred kernel weight of maize was highest under Straw + LGM (34.8 g), which was followed by Straw (33.4 g). In the third rotation cycle, seed setting rate of sunflower was highest under Straw (53.9%) and lowest under CK (41.9%).

The yield sustainability index (YSI) of maize under Straw + LGM was significantly higher than under Straw and CK, while the YSI index of sunflower under Straw was significantly greater than Straw + LGM and CK (Table 3). For maize, Straw + LGM had the highest YSI, which was 6.7% and 9.1% higher than that under CK and Straw, respectively. For sunflower, Straw treatment had the highest YSI, which was 19.4% and 21.3% larger relative to CK and Straw + LGM treatments, respectively.

### 2.2. Nutrient Use Efficiency, Nutrient Uptake and Nutrient Harvest Index

**Figure 2:**
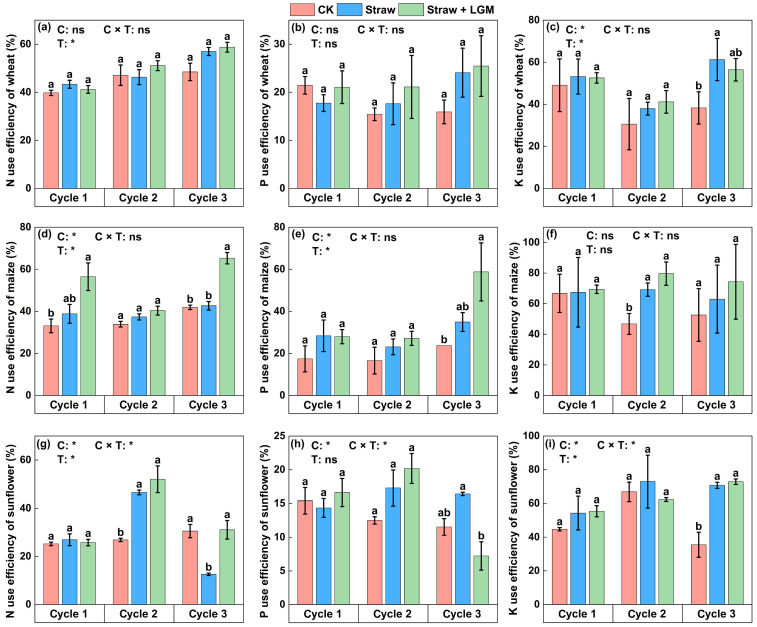
The nutrient utilization efficiency of wheat (**a**–**c**), maize (**d**–**f**) and sunflower (**g**–**i**) in spring wheat–maize–sunflower rotation system. Bars represent standard errors. The different lowercase letters indicate significant differences between the fertilization treatments at the *p* = 0.05 level. N: nitrogen; P: phosphorus; K: potassium. CK: chemical fertilizer alone; Straw: chemical fertilizer with returned straw; Straw + LGM: chemical fertilizer with returned straw and leguminous green manure; C: rotation cycle; T: treatment; * means *p* < 0.05; ns means *p* > 0.05.

For wheat, crop rotation cycle significantly affected N use efficiency (Figure 2a, *p* < 0.05). Different organic amendment treatments significantly affected K use efficiency in wheat in the third rotation cycle (Figure 2d, *p* < 0.05). Specifically, K use efficiency was the highest under Straw (61.3%) in the third rotation cycle, followed by Straw + LGM (56.4%) and CK (38.3%). For maize, both crop rotation cycle and organic amendment treatments significantly affected the N and P use efficiency (Figure 2d,e). In the third rotation cycle, N use efficiency was significantly higher under Straw + LGM as compared to Straw and CK, and P use efficiency among treatments was in descending order as follows: Straw + LGM (58.8%) > Straw (34.9%) > CK (23.7%). For sunflower, crop rotation cycle, different organic amendment treatments and their conformity effects all significantly affected nutrient use efficiency (Figure 2g–i, *p* < 0.05). In the second rotation cycle, nitrogen use efficiency was significantly higher under Straw (46.5) and Straw + LGM (52.0%) than that under CK (26.9%), and in the third rotation cycle, N use efficiency was significantly higher under Straw + LGM (31.8%) and CK (30.5%) compared with Straw (12.6%); P use efficiency was significantly greater under Straw than Straw + LGM. Sunflower showed higher K use efficiency of 70.7% (Straw) and 72.8% (Straw + LGM) in the third crop rotation.

**Figure 3:**
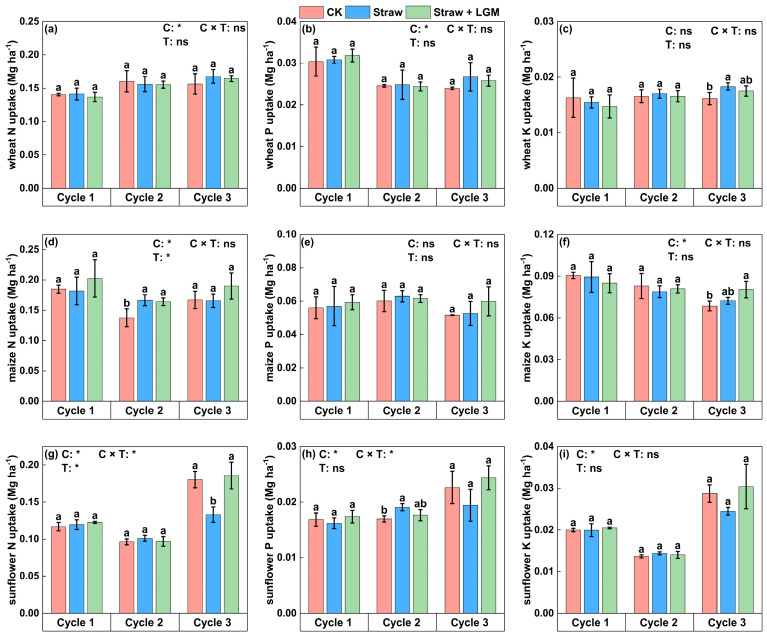
The nutrient uptake of wheat (**a**–**c**), maize (**d**–**f**) and sunflower (**g**–**i**) in spring wheat-maize-sunflower rotation system. Bars represent standard errors. The different lowercase letters indicate significant differences between the fertilization treatments at the *p* = 0.05 level. N: nitrogen; P: phosphorus; K: potassium. CK: chemical fertilizer alone; Straw: chemical fertilizer with returned straw; Straw + LGM: chemical fertilizer with returned straw and leguminous green manure; C: rotation cycle; T: treatment; * means *p* < 0.05; ns means *p* > 0.05.

Crop rotation significantly affected wheat N and P uptake (Figure 3). In the third crop rotation cycle, wheat K uptake was significantly increased under Straw (0.02 Mg ha^−1^) than Straw + LGM (Figure 3c; *p* < 0.05). Crop rotation significantly affected maize N and K uptake, and in the third rotation cycle, maize uptake of K was significantly higher under Straw + LGM (0.08 Mg ha^−1^) than Straw (0.07 Mg ha^−1^). The uptake by sunflower of N, P and K was greater in the third rotation cycle than in the first two cycles. Specifically, in the third crop rotation cycle, sunflower N uptake was significantly higher under Straw + LGM (0.18 Mg ha^−1^) and CK (0.18 Mg ha^−1^) than Straw (0.13 Mg ha^−1^), and for P and K uptake, although not statistically different, the same trend was observed: Straw + LGM ≈ CK > Straw.

**Figure 4:**
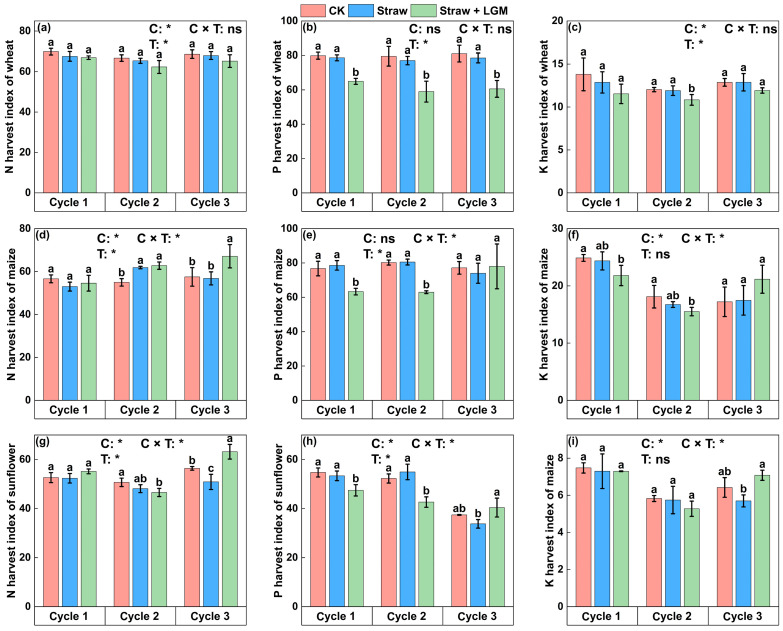
The nutrient harvest index of wheat (**a**–**c**), maize (**d**–**f**) and sunflower (**g**–**i**) in three rotation cycles. Bars represent standard errors. The different lowercase letters indicate significant differences between the fertilization treatments at the *p* = 0.05 level. N: nitrogen; P: phosphorus; K: potassium. CK: chemical fertilizer alone; Straw: chemical fertilizer with returned straw; Straw + LGM: chemical fertilizer with returned straw and leguminous green manure; C: rotation cycle; T: treatment; * means *p* < 0.05; ns means *p* > 0.05.

The wheat N harvest index did not show any significant difference among the three cycles (Figure 4; *p* > 0.05), and the P harvest index was the lowest under Straw + LGM among the three cycles. For maize, the P harvest index under Straw and Straw + LGM (61.9 and 62.8, respectively) was significantly higher than under CK in the second rotation cycle, while it was higher under Straw + LGM (67.1) than under Straw and CK in the third rotation cycle. The P harvest index of maize under Straw + LGM was significantly lower than under Straw and CK in the first two rotations cycles, and there was no significant difference in the P and K harvest indexes in the third cycle. N harvest index of sunflower in the third cycle was in the following descending order: Straw + LGM (58.5) > CK (56.3) > Straw (50.9). The P harvest index of sunflower was the lowest in the first two cycles under Straw + LGM. In spite of that, in the third cycle, it was significantly greater under Straw + LGM (40.4) than under Straw and CK. Likewise, the K harvest index of sunflower in the third cycle was significantly greater under Straw + LGM (7.08) than Straw and CK.

### 2.3. Grain Yield in Relation to Nutrient Use Efficiency and the Nutrient Harvest Index

N use efficiency was significantly correlated with yield only under the LGM return (Figure S1, *p* < 0.001, R^2^ = 0.74). P use efficiency and yield were significantly correlated under different fertilizer treatments, but the correlation was higher under Straw and Straw + LGM. K use efficiency was significantly correlated with yield only under leguminous green manure return (Figure S1, *p* = 0.03, R^2^ = 0.13).

**Figure 5:**
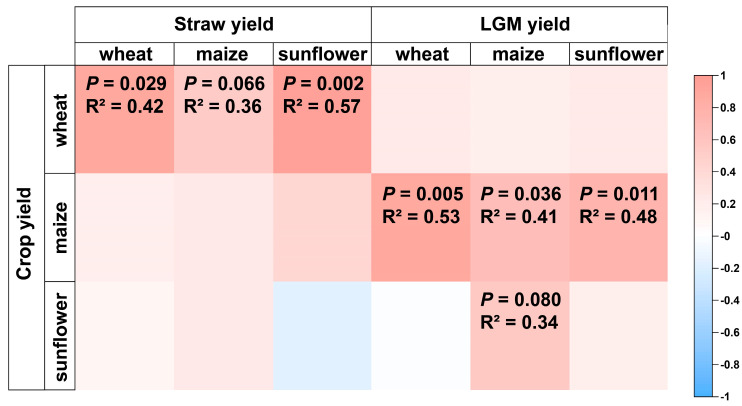
Spearman analysis of straw, leguminous green manure yield and crop yield under the different fertilizer treatments. LGM: leguminous green manure.

**Figure 6:**
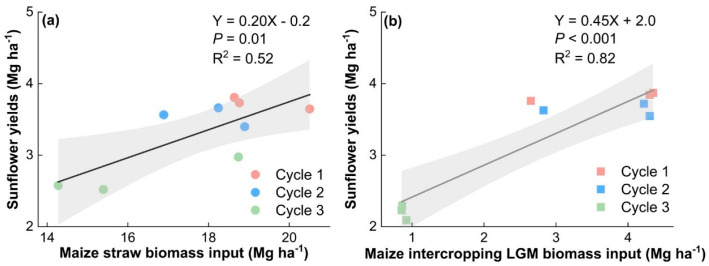
Regression analysis of maize straw biomass (**a**) and leguminous green manure biomass (**b**) inputs with yield of later crops.

Correlation analysis revealed that inputs of straw were found to be significantly correlated with wheat yield, and inputs of LGM were significantly correlated with maize and sunflower yield (Figure 5). Additionally, maize straw inputs explained up to 0.52 of sunflower yields, while LGM inputs explained up to 0.82 of sunflower yields (Figure 6).

### 2.4. Economic Benefits

Economic benefits were assessed between crop rotation and fertilizer treatments (Table 4). In all three crops, the cost was highest under Straw + LGM and lowest under CK. The cost of wheat was 1380.1 under CK, 1485.1 under Straw and 1915.6 (USD ha^−1^) under Straw + LGM. In maize season, the cost of Straw + LGM was the greatest at 1876 (USD ha^−1^), which was 29.8% and 21.0% higher than CK and Straw, respectively. In sunflower season, the cost of Straw + LGM was the highest at 1876 (USD ha^−1^), which was 29.8% and 21.0% higher than CK and Straw, respectively. In the first rotation cycle, wheat, maize and sunflower had the highest net income under CK with 1657.4, 3038 and 1645.7 (USD ha^−1^), respectively. In the second rotation cycle, wheat had the highest net income of 1563.3 USD ha^−1^ under CK, which was 5.6% and 45.1% higher than the Straw and Straw + LGM treatment, respectively. Similarly, in the second rotation cycle, maize had the highest net income of 3638.2 USD ha^−1^ under CK, which was 11.3% and 2.3% higher than the Straw and Straw + LGM treatments, respectively. In the second rotation cycle, the highest net income of sunflower was under Straw (1621.4 USD ha^−1^), which was 3.0% and 17.8% higher than the CK and Straw + LGM treatments. In the third rotation cycle, for wheat the Straw had the highest net income benefit of 1749.5 USD ha^−1^, while the Straw + LGM had the lowest at 1287.6 USD ha^−1^. Conversely, for maize, the Straw + LGM had the highest net income benefit of 4028.1 USD ha^−1^, which was 15.8% and 16.6% greater than the CK and Straw treatments. For sunflower, Straw had the highest net income benefit of 1688.3 USD ha^−1^ and Straw + LGM the lowest with 784.9 USD ha^−1^.

## 3. Discussion

### 3.1. Impacts of the Combined Application of Leguminous Green Manure and Straw on Crop Yields

Maintaining crop yield is the most essential outcome of sustainable agriculture practices [32]. Our results are consistent with previous studies in which the application of LGM has been found to increase maize yield by about 713 kg ha^−1^ [33]. The positive effects of LGM on crop yield have been reported in numerous studies, and the reasons may be as follows: (1) The long-term application of chemical fertilizers could lead to soil acidification and even to a reduction in biological N fixation, lower soil fertility and reduced crop yields, particularly in saline–alkali soil. However, the application of LGM could potentially reduce negative effects of soil salinity in the Hetao Irrigation District and increase microbial activity, thereby increasing crop yields under saline soils [14,29,34]. (2) Straw return could increase the accumulation of soil organic carbon [17]; LGM plus straw promotes the degradation of straw compared to straw returned to the field. LGM could also release considerable N in time for the next season’s crop [35], which further increases crop yields [36,37,38]. (3) The inclusion of LGM crops as part of the rotation in traditional cropping systems could increase the accumulation of soil organic carbon and nitrogen in all the soil aggregate size fractions and also contribute to improved crop growth [35]. Nevertheless, our data showed that although LGM and straw promoted increased sunflower yields in the first two cycles, sunflower yields decreased in the third crop rotation cycle (Figure 1c; *p* < 0.05), which is inconsistent with our first hypothesis. As one of the most deeply-rooted crops, sunflower is adept at utilizing subsoil nutrients and water; thus, long-term tillage may result in sunflowers depleting nutrients from the soil substrate, leading to reduced yields [39]. At Colby, Kansas, research suggests that a winter wheat–corn–sunflower–grain sorghum–fallow rotation system is worth considering for higher yield due to the lower available soil water that exists when sunflower is the prior crop in rotation [40]. Furthermore, through a comprehensive model evaluation, a study revealed that spacing sunflower plantings three or more years apart is optimal for achieving maximum yield and crop health [41].

The YSI index is a reliable parameter for measuring whether a system can produce consistently and stably (Table 3). The YSI index was significantly highest for maize and lowest for sunflower under Straw + LGM. The impact of LGM on crops could last for 2–3 seasons [42,43,44,45]. Additionally, the crop previous to maize was LGM, which means the return of LGM was higher than for the other crops during the maize season, contributing to the positive effect of LGM on the crop [21,46] (Table S2). Hence, maize has the highest yield sustainability index of the three crops.

### 3.2. Grain Yield in Relationship to Nutrient Use Efficiency and Nutrient Harvest Index

Nutrient use efficiency is an important measurement of the sustainability of crop production systems [7]. Despite the fact that straw return could enhance crop yield, it did not improve the nutrient use efficiency of maize (Figure 2d–f; *p* < 0.05); this is because straw is more difficult to decompose and the nutrients more difficult to release for the following crops. According to Palm (1995), about 80% of the nutrients are released during decomposition of organic residues, but less than 20% are absorbed by the crop [47]. Consistent with our second hypothesis, the application of LGM enhanced the nutrient use efficiency of the crop (Figure 2). The effect of LGM (as high-quality organic residues) as a fertilizer on improving plant nutrient use efficiency has been previously observed [48,49]. Legume residues are considered as superior quality materials due to their low C:N ratio, low content of lignin and polyphenols. These characteristics facilitate rapid nutrient release during the decomposition process. [50,51]. In addition, it has been found that the addition of LGM accelerates the decomposition of straw by stimulating microbial growth [46,52].

Linear regression further revealed that crop N and K use efficiency under LGM application was significantly correlated with crop yield, implying that LGM promotes nutrient utilization in crops (Figure S1). Only P use efficiency was significantly correlated with yield under all three different treatments, whereas N use efficiency and K use efficiency were significantly correlated only under Straw + LGM. In the third cropping cycle, the N harvest index for both maize and sunflower was significantly lower under straw treatment than under LGM, so even though straw return could increase crop yield to some extent, no statistical correlation was observed as it did not improve nutrient use efficiency. This implies that the application of LGM can enhance crop yield by improving N or K use efficiency. Accordingly, increasing the nutrient use efficiency in LGM application results in higher biomass production, which in turn leads to more crop residues in the cultivation system [53,54]. Thus, through degradation and mineralization of organic substances, more plant-available nutrients will be provided for subsequent crops. This in turn can lead to an increase in the populations of soil microorganisms and their activity to promote nutrient use efficiency [55].

Resource allocation to reproduction is a critical trait for plant health and is called the nutrient harvest index in the agricultural context. It provides an indication of how efficiently the plant utilizes acquired N, P and K for grain production. The uptake of N, P and K by both maize and sunflower was greater (statistically or non-statistically) under Straw + LGM than Straw in the third cycle (Figure 3). But for wheat, there was no significant difference in N uptake by grain under different fertilizer treatments. This may be for the following reasons: (1) the return of LGM stimulates decomposition of the straw and therefore nutrient uptake by the crop [46,52], and (2) intercropping of the LGM with maize and sunflower promotes nutrient uptake [20]. The ability of crop rotation to enhance plant nutrient uptake to facilitate maize yield has been reported in previous studies [56]. In our study, we found that crop rotation promoted N, P and K uptake by sunflower seeds: the uptake of N, P and K was significantly greater in the third cycle than in the first two cycles, and this is consistent with the nutrient harvest index of sunflower (Figure 4). In the present study, increasing the N harvest index of maize (94%) and sunflower (66%) under Straw + LGM compared to Straw and CK in the third crop rotation reflected N accumulation by the crop (Figure 4), which is not only provided by N fertilizer but also by biological N fixation practices. Differences in the observed N harvest index among the three crops in rotation could possibly also have resulted from differences in fertilizer management. The slower mineralization of the organic matter in the incorporated LGM probably synchronized soil N supply and crop N demand, thereby primarily affecting the N harvest index. A high N harvest index is associated with efficient utilization of N and increased partitioning of N to the grain [57]. Furthermore, the increase of the N harvest index will also increase the grain quality of cereals [58]. Our study found a positive correlation between yield and N harvest index in the Straw vs. CK analysis (Figure S2), which is consistent with previous studies; there is a significant correlation between the N harvest index and yield, and increasing a crop’s N harvest index can lead to yield increases [57]. In contrast, there was no statistically significant correlation between N harvest index and yield for straw + LGM (Figure S2) because LGM could not enhance sunflower yield in the third crop cycle in the wheat–maize–sunflower rotation system, although it could improve the N harvest index of sunflower. Fixed N acts as a source of storage compounds that support N accumulation in grains; in other words, LGM maintained the quality of sunflower seeds although it did not maintain sunflower yield.

Linear regression further revealed a significant relationship between LGM and straw application on maize subsequence crop (sunflower) yield in the wheat–maize–sunflower rotation system (Figure 5). Each additional input of 1 Mg ha^−1^ of straw and LGM increased maize subsequence crop yield by 0.2 and 0.45 Mg ha^−1^, respectively. Therefore, Straw + LGM was more important to the yields of sunflower in the following year in rotation than the sole application of straw. LGM application could replenish organic N reserves which are important for long-term soil productivity [59]. Furthermore, the fixed atmospheric N is released into the soil in mineral N form with the decomposition of LGM. Thus, the lower sunflower yields in the third cycle may be due to lower LGM inputs (Figure 5 and Figure 6; Table S2), and we recommend increasing the input of LGM to support higher sunflower yields. In addition, The R^2^ of Straw is lower, which implies higher variability around the regression line. For instance, the application of straw alone may not directly contribute additional nutrients or organic matter to the soil compared to straw with LGM. Improved farmland productivity and ecological services were observed through long-term green manure cultivation and incorporation [60]. Therefore, we surmise that reliance solely on straw inputs might make the crop more vulnerable to external factors like weather variations or soil nutrient deficiencies. In contrast, LGM not only provides organic matter but also introduces nutrients, including nitrogen, which can potentially mitigate the impact of external factors by improving soil fertility and enhancing crop resilience [61].

### 3.3. Implications for Agricultural Sustainable Development

The increasing cost of agricultural inputs and fluctuating prices of products contributed to the variations in the final economic performance of the different cropping systems. Maize was better in terms of economic performance compared with the other two crops in the second and third crop rotation systems (Table 3), which also indicates that maize could secure net benefits to farmers. However, in the case of chemical fertilizers only or chemical fertilizers plus straw, even if the yield is not high, it will result in a profitable outcome. LGM application, with its high labor requirements for field management practices (such as sowing and cultivation), may become too costly, especially since, with the development of industry, synthetic fertilizers are inexpensive [62]. Evidence suggests that due to the weed suppression as well as higher N fixation of LGM, the N contribution from the LGM may offset the need for N fertilizer or herbicide [59], and if the cost of fertilizer and/or herbicides were to increase substantially, the economic benefit from LGM may become more crucial. In this experiment, to control the variables we used the same field management practices under three different treatments. Hence, LGM application may not be economically justified without the provision of multiple services such as nutrient supply, weed control and improvement of soil characteristics for crop production, among others. 

Over-application of chemical fertilizer may lead to a reduction in crop nitrogen use efficiency [46]. A previous study found that lower nitrogen use efficiency in developing countries is associated with lower government economic subsidies [63], and an assessment of China’s strategy to improve N use efficiency reported that socioeconomic conditions must be improved to use nitrogen more efficiently in crop production [64], for example by phasing out N fertilizer subsidies and encouraging the adoption of best management practices [8]. As an important source of N, LGM can contribute to sustainable agricultural development and maintain or even increase the yield of the main crops [65]. The Hetao Irrigation District is the main grain-producing area in China [29], and in order to ensure food security and promote the economic income of local farmers, the role of LGM in agricultural production activities should be emphasized. The traditional style of agricultural research and technology transfer may poorly suit the development of LGM approaches to crop production [66]. Economic competitiveness of LGM may thus require delivery of multiple services to improve economic returns from the use of LGM, such as provision of biologically fixed N, pest and weed control, increase of soil organic matter and reduction of soil erosion or agrochemical loss. 

Overall, both Straw and Straw + LGM increased yields, but only LGM application plus straw improved crop N use efficiency and harvest index. Hence, LGM has a greater potential to contribute to global food security and the sustainable development of agriculture [38]. Our research highlights the importance of LGM application and diversified crop rotations to improve nutrient use efficiency and maintain yields, and we therefore recommend that the government should subsidize LGM application to increase the use of LGM.

## 4. Materials and Methods

### 4.1. Site Description

**Figure 7:**
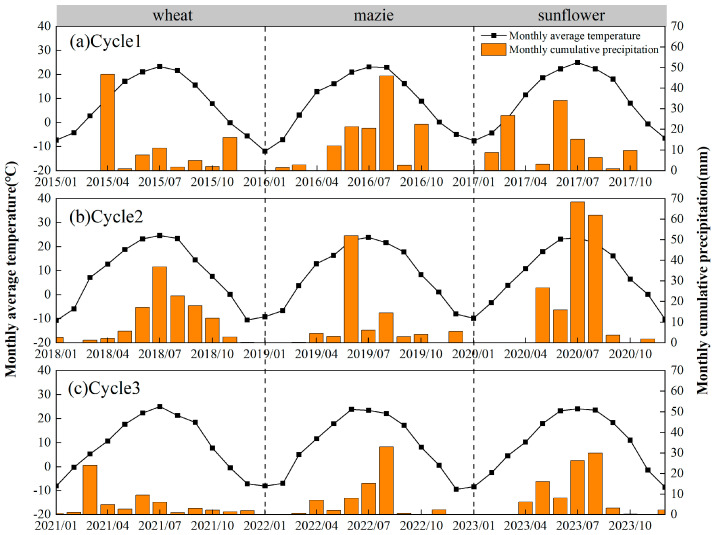
Precipitation and temperature at the experimental site in 2015–2023.

This study was conducted at the Yuanzi Drainage Experimental Station (40°90′ N, 107°17′ E, altitude 1035 m), Academy of Agricultural & Animal Husbandry Sciences, Bayannur City, Inner Mongolia, China, and the experiment was conducted from March 2015 to October 2023. The region has a temperate, dry, and continental monsoon climate. The annual precipitation from 2004 to 2024 was 188 mm (mainly falling between May and September), and the annual evaporation was 2030–3180 mm (Figure 7). There were 3100–3300 h of sunshine and 126 days without frost, on average, per year [29]. The soil was silt loam with soil organic matter content of 13.0 g kg^−1^ (0–20 cm). Available nitrogen (N) concentration was 0.073 g kg^−1^; available phosphorus (P) concentration was 0.026 g kg^−1^; exchangeable potassium (K) concentration was 0.130 g kg^−1^; soil total water-soluble salt was 0.58 g kg^−1^; and the soil pH was 8.8. The detailed meteorological data are shown in Figure 7.

### 4.2. Experimental Design

**Figure 8:**
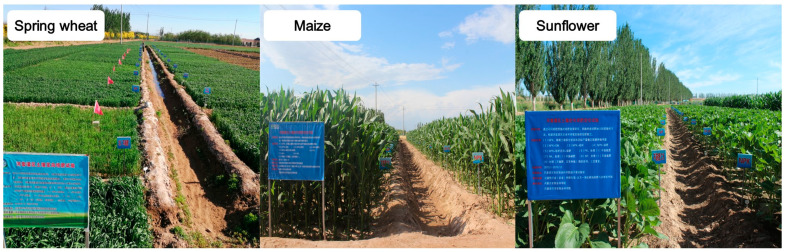
Field experiment of wheat–maize–sunflower rotation system.

Nine years of field trials in a randomized block design were conducted using the wheat–maize–sunflower rotation system from 2015 to 2023 (Figure 8). There were 3 treatments in total and each treatment included 3 replications. Each plot area was 5 × 8 = 40 m^2^. The treatments used in this study included: CK (chemical N, P and K fertilizers only); Straw (chemical N, P and K fertilizers and returned straw); Straw + LGM (chemical N, P and K fertilizers and returned straw, combined with LGM). Planting was as follows: in the first year, LGM (hairy vetch) with a spring wheat–vetch rotation; maize/vetch intercropping in the second year; and sunflower/vetch relay intercropping in the third year (Figure 8). The straw return was applicable to all three types of crops including wheat, maize and sunflower. LGM crops were grown until the full-bloom stage, and then plants were cut into 2–3 cm pieces before being incorporated back into the field using a rotary tillage machine in the next year. Immediately after harvesting the spring wheat, rotary ploughing was undertaken to hasten the planting of hairy vetch. Maize and sunflower were planted in large and small rows. The amount of straw/green manure input for each crop was determined by the straw biomass/green manure biomass of last year’s crop. Biomass of straw and LGM is shown in Tables S1 and S2. The average nutrient contents in the LGM and straw are shown in Table S1. Specifically, the average nutrient contents in the LGM, including N, P_2_O_5_ and K_2_O, were 3.8%, 0.35% and 3.03%, respectively. The rate of N fixation in LGM monoculture and intercropping was 64.1% and 60.1%, respectively. The data were derived from research on N fixation by LGM [1] since the soil properties and pH of our experiment area are similar to that research. The average nutrient contents in the spring wheat straw, including N, P_2_O_5_, and K_2_O, were 0.61%, 0.15%, and 1.37%, respectively (Table 5). The average nutrient contents in the maize straw, including N, P_2_O_5_ and K_2_O, were 0.65%, 0.11% and 1.62%, respectively. The average nutrient contents in the sunflower straw, including N, P_2_O_5_ and K_2_O, were 0.69%, 0.11% and 2.33%, respectively.

### 4.3. Field Management

The LGM was Turkmen hairy vetch, seeded at 75 kg ha^−2^ in late September after the wheat harvest. The maize was planted in large (70 cm) and small rows (40 cm), and LGM was planted between the large rows of maize, with a sowing rate of 45 kg ha^−2^, and harvested during the early flowering stage of LGM, and then tipped in the rows. The sunflower was planted in large (80 cm) and small rows (40 cm), furrowed and fertilized with mulch. In late April, the sunflower rows were planted with LGM at a rate of 75 kg ha^−2^. The sunflower was then sown in late May, and LGM was harvested during the early flowering stage and tilled in situ in the inter-row area. 

The spring wheat variety used was Yongliang 4 at a rate of 375 kg seed ha^−1^. Wheat was sown with a row spacing of 15 cm. The maize variety used was Ximeng 568, sown with a plant distance of 24 cm within a row, at a density of 75,000 plants ha^−1^. The sunflower variety used was SH363 sown with a plant distance of 50 cm within a row, at a density of 33,000 plants ha^−1^. Both maize and sunflower were grown with plastic film mulching. Plough tillage was performed before irrigation the previous autumn, and the land was tilled to a depth of 20 cm with a rotavator before sowing. P fertilizers were all applied as seed fertilizer; 70% of K fertilizers were applied as seed fertilizer and 30% were applied in combination with irrigation (at the nodulation stage for wheat and maize and at the bud stage for sunflowers); 30% of N fertilizers were applied as seed fertilizer and 70% were applied in combination with irrigation (at the nodulation stage for wheat and maize and at the bud stage for sunflowers). The fertilization rates in the wheat field were 225 kg N ha^−1^, 120 kg P_2_O_5_ ha^−1^ and 130 kg K_2_O ha^−1^; maize and sunflower were fertilized with 270 kg N ha^−1^, 120 kg P_2_O_5_ ha^−1^ and 130 kg K_2_O ha^−1^. The fertilizers were urea, diammonium phosphate and K sulfate, respectively. The same amount of chemical fertilizer was applied in the three different treatments. The experimental plots were all irrigated with Yellow River water using border irrigation, with 1875 m^3^ ha^−1^ for wheat, 2250 m^3^ ha^−1^ for maize and 1125 m^3^ ha^−1^ for sunflower. The sowing and harvest dates and other field managements practices are provided in Table S3.

### 4.4. Determination of N, P and K Uptake by Crops

In the harvest period of spring wheat, three representative sample points were selected in each plot; each sample point was 3.14 m^2^ (circles with a diameter of 1 m). Maize and sunflower were measured by the total yield of each plot, and the yield was determined by threshing after natural air drying. Ten plants were randomly selected from each plot to determine yield components such as spike length, number of grains in a spike, 100 kernel weight/1000 kernel weight, etc., and to record the yield of hairy camas in the LGM plot and the amount of straw returned to the field in the straw-return plot.

At crop maturity, 10 wheat plants (stalks, seeds, leaves), 5 maize plants (stalks, seeds, leaves) and 5 sunflower plants (stalks, seeds, discs) were taken from each treatment and placed in mesh bags by organ, respectively, and oven-dried at a constant 65–70 °C. Additionally, dried sub-samples were ground and digested using a concentrated H_2_SO_4_ and H_2_O_2_ solution, and subjected to the micro-Kjeldahl procedure to measure N concentrations. In addition, total P was determined using the molybdenum–antimony colorimetric method, while total K was determined by flame photometry.

### 4.5. Sampling and Laboratory Measurements

Soil samples were collected with a 5 cm diameter corer (inner diameter: 4 cm) from 0–20 cm and 20–40 cm in each experimental plot after harvesting wheat, maize and sunflower. The samples were stored in sealed plastic bags and taken back to the laboratory as soon as possible. The air-dried soil samples were then ground to pass a 0.25 mm sieve for soil organic matter and total nitrogen determination using the K_2_Cr_2_O_7_–H_2_SO_4_ wet oxidation and the Kjeldahl method, respectively. Available P was determined by sodium bicarbonate leaching and the molybdenum–antimony colorimetric method; exchanged K by ammonium acetate leaching and the flame photometric method; and soil pH by the potentiometric method (water–soil ratio 2.5:1).

### 4.6. Data Calculations

To compare the performance of the three cropping systems, the grain yield of maize and sunflower was converted to wheat equivalent yield (Mg/ha) as described previously [11]:

(1) $$\mathrm{Wheat}\;\mathrm{equivalent}\;\mathrm{yield}=\frac{Pnon-wheat}{Pwheat}\times \mathrm{Ynon-wheat},$$

where Pnon-wheat is the price of non-wheat crops, P wheat is the price of wheat, and Ynon-wheat is the yield of non-wheat crops. The average price for wheat, maize and sunflower was 0.45, 0.34 and 0.98 USD kg^−1^ during the study period.

Economic analysis was conducted to determine the average economic feasibility of the cropping systems. The total inputs, including seeds, fertilizers, irrigation (electricity), machinery and labor, were calculated on the basis of local conditions (Table S3). Gross income was estimated according to the average price and yield. Net income was calculated by deducting total costs from the gross income.

The yield increase rates were calculated as follows:

(2) $$\mathrm{Yield}\;\mathrm{increasing}\;\mathrm{rate}\;(\%)=\frac{Y-Yo}{Yo}\times 100\%,$$

where *Y* is the crop yield under different fertilizer treatment (kg ha^−1^) and *Yo* is the crop yield without fertilizer.

The nutrient use efficiency was calculated as follows [62]:

(3) $$\mathrm{Nutrients}\;\mathrm{use}\;\mathrm{efficiency}\;(\%)=\frac{U-Uo}{F}\times 100\%,$$

where *F* is the total fertilizer input (kg ha^−1^), *U* is the total amount of nutrient (N, P and K) taken up by above-ground plant biomass at maturity in soils with fertilizer, *Uo* is the total amount of nutrient taken up by above-ground plant biomass at maturity in soils without fertilizer.

The nutrient harvest index (NHI) was calculated as [62]:

(4) $$\mathrm{NHI}\;\left(\%\right)=\frac{Gy}{Gy+Ly+Sy}\times 100\%,$$

where *Gy* is the grain nutrients (N, P and K) yield (kg ha^−1^), *Ly* is the leaf nutrients yield and *Sy* is stem nutrients yield.

The yield sustainability index (YSI) was calculated to describe the yield stability. YSI was calculated as [67]:

(5) $$\mathrm{YSI}=\frac{Y_{mean-Y_{std}}}{Y_{max}}\times 100\%$$

where *Y_mean_* is the mean crop grain yield, *Y_std_* is the standard deviation of the grain yield across the entire duration (years), and *Y_max_* is the maximum observed grain yield.

### 4.7. Data Analysis

The raw data were collated using Excel (2019; Microsoft, Redmond, WA, USA) and nutrient input was calculated. The statistical analysis was performed separately for each rotational year according to treatment factors (CK, straw and straw + CK) by using one-way analysis of variance (one-way ANOVA) and least significance difference (LSD) in SPSS 22.0 software (IBM, New York, NY, USA). Two-way analysis of variance (ANOVA) was used to reveal the effects of the rotation system and treatment on crop yields. The significance level was set at *p* < 0.05. Linear regressions of the nutrient harvest index and nutrient use efficiency to yield were calculated and embellished using Origin Pro (Version 2024; OriginLab Corporation, Northampton, MA, USA).

## 5. Conclusions

Our results showed that the application of hairy vetch as leguminous green manure with straw in diversified crop rotation increased maize yield compared to chemical fertilization and straw return. The combined application of straw and leguminous green manure increased nutrient use efficiency (N and P) and the nutrient recovery index for wheat, maize and sunflower, and subsequently contributed to the improvement in crop yields. Although sunflower yield did not increase in the third rotation, enlarging the intercropping area of leguminous green manure with maize could maintain and/or promote sunflower yields in long-term agricultural production activities. Straw return led to an increase of net income from wheat and sunflower. In addition, LGM application did not increase economic income in the short term; however, it can contribute to the sustainability of agricultural systems. Hence, planting leguminous green manure in the wheat–maize–sunflower diversified cropping rotation in the Hetao District in Northwest China could be viewed as a potential sustainable agronomic practice to promote yield production.

## Figures and Tables

**Table 1:** The yield increase rate of Straw and Straw + LGM.

Rotation Cycle	Treatment	Wheat	Maize	Sunflower
Cycle 1 (2015–2017)	CK	1.77 ± 0.21 a	0.61 ± 0.10 a	0.15 ± 0.04 a
	Straw	1.80 ± 0.10 a	0.63 ± 0.20 a	0.17 ± 0.01 a
	Straw + LGM	1.80 ± 0.10 a	0.64 ± 0.10 a	0.20 ± 0.05 a
Cycle 2 (2018–2020)	CK	5.53 ± 0.78 a	1.63 ± 0.65 a	0.17 ± 0.06 a
	Straw	1.77 ± 2.97 b	0.50 ± 0.87 a	0.17 ± 0.12 a
	Straw + LGM	5.67 ± 1.00 a	1.80 ± 0.50 a	0.27 ± 0.06 a
Cycle 3 (2021–2023)	CK	2.00 ± 0.10 b	1.10 ± 0.10 b	0.37 ± 0.21 a
	Straw	2.30 ± 0.10 a	1.13 ± 0.15 b	0.67 ± 0.15 a
	Straw + LGM	2.27 ± 0.06 a	1.53 ± 0.15 a	0.40 ± 0.00 a

Note: Values are means ± standard errors. The different lowercase letters indicate significant differences between the fertilization treatments at the *p* = 0.05 level. CK: chemical fertilizer alone; Straw: chemical fertilizer with returned straw; Straw + LGM: chemical fertilizer with returned straw and leguminous green manure.

**Table 2:** The yield components of wheat, maize and sunflower.

Rotation Cycles	Treatments	Wheat			Maize			Sunflower		
		Spike Number (m^−2^)	Grain Number (Spike^−1^)	1000-Grain Weight (g)	Ear Number (m^−2^)	Kernel Number (Ear^−1^)	100-Kernel Weight (g)	Head Number (m^−2^)	Seed Setting Rate (%)	1000-Seed Weight (g)
Cycle 1 (2015–2017)	CK	719.9 ± 2.7 a	34.7 ± 1.2 a	53.9 ± 1.8 a	7.4 ± 0.3 a	41 ± 0.9 b	35.1 ± 0.7 a	3.01 ± 0.02 a	88.7 ± 3.4 a	177.1 ± 9 a
	Straw	705 ± 27.8 a	33.1 ± 1.7 a	54.3 ± 0.9 a	7.4 ± 0.1 a	43 ± 0.9 a	36.9 ± 0.3 a	3.02 ± 0.02 a	88.7 ± 3.9 a	180.8 ± 0.8 a
	Straw + LGM	716.7 ± 4.4 a	33.5 ± 0.6 a	51.3 ± 2.7 a	7.3 ± 0.2 a	41 ± 0.9 b	35.1 ± 0.7 a	3.08 ± 0.08 a	90.2 ± 4 a	190.3 ± 5.3 a
Cycle 2 (2015–2017)	CK	727.9 ± 4.1 a	36.4 ± 1.9 a	46.8 ± 0.7 a	7.2 ± 0.3 a	42.5 ± 0.9 a	33.4 ± 0.2 b	3.00 ± 0.12 a	83.6 ± 0.5 a	234.2 ± 12.3 a
	Straw	694.2 ± 28.6 b	34.4 ± 1.3 a	43 ± 1.7 a	7.3 ± 0.1 a	40.4 ± 0.3 a	36.6 ± 0.6 a	2.93 ± 0.08 a	83.6 ± 1.8 a	240.9 ± 1.2 a
	Straw + LGM	719.1 ± 11.6 a	34.9 ± 0.6 a	46.7 ± 1.5 a	7.4 ± 0.1 a	42.1 ± 0.3 a	35.8 ± 0.8 a	2.9 ± 0.04 a	82.4 ± 1.1 a	235.9 ± 3.5 a
Cycle 3 (2015–2017)	CK	723.8 ± 19 a	35.4 ± 1.6 b	49.6 ± 2.2 a	7.1 ± 0.1 a	37.9 ± 1.1 a	32.8 ± 0.5 b	2.63 ± 0.03 a	41.9 ± 2.3 b	274.1 ± 5.6 a
	Straw	701.9 ± 40.8 a	38.7 ± 0.9 ab	50.4 ± 0.4 a	7 ± 0.1 a	38.2 ± 1.2 a	33.4 ± 0.5 ab	2.48 ± 0.03 a	53.9 ± 3.4 a	273.7 ± 2.5 a
	Straw + LGM	711.3 ± 18.5 a	41.2 ± 1.3 a	51.2 ± 2.5 a	7.2 ± 0.2 a	39.3 ± 1.7 a	34.8 ± 0.3 a	2.61 ± 0.02 a	44.9 ± 1.6 ab	269.3 ± 1.7 a
Cycle		*	*	ns	*	*	*	*	*	*
Treatment		ns	ns	ns	ns	ns	*	ns	ns	ns
C × T		ns	ns	ns	ns	ns	*	ns	ns	ns

Note: Values are means ± standard errors. The different lowercase letters indicate significant differences between the fertilization treatments at the *p* = 0.05 level. CK: chemical fertilizer alone; Straw: chemical fertilizer with returned straw; Straw + LGM: chemical fertilizer with returned straw and leguminous green manure; C: rotation cycle; T: treatment; * means *p* < 0.05; ns means *p* > 0.05.

**Table 3:** The yield sustainability index in the wheat–maize–sunflower rotation system.

	Wheat	Maize	Sunflower
CK	0.95 ± 0.03 a	0.83 ± 0.06 b	0.62 ± 0.04 b
Straw	0.91 ± 0.01 a	0.86 ± 0.01 b	0.74 ± 0.07 a
Straw + LGM	0.93 ± 0.01 a	0.91 ± 0.02 a	0.61 ± 0.03 b

Note: Values are means ± standard errors. The different lowercase letters indicate significant differences between the fertilization treatments at the *p* = 0.05 level. YSI: yield sustainability index; CK: chemical fertilizer alone; Straw: chemical fertilizer with returned straw; Straw + LGM: chemical fertilizer with returned straw and leguminous green manure.

**Table 4:** Cost, gross income and net income of wheat, maize and sunflower under different treatments across the three cycles of the wheat–maize–sunflower rotation system.

Rotation Cycle	Spring Wheat			Maize			Sunflower		
	CK	Straw	Straw + LGM	CK	Straw	Straw + LGM	CK	Straw	Straw + LGM
**Cost (USD ha^−2^)**									
	1380.1	1485.1	1915.6	1445.5	1550.5	1876	1445.5	1550.5	1876
**Gross income (USD ha^−2^)**									
Cycle1 (2015–2017)	3037.4 ± 84.1	3068.8 ± 51.3	3064.3 ± 75.6	4483.5 ± 117.8	4545.2 ± 630.6	4574.6 ± 67.5	3091.2 ± 29.8	3133.2 ± 67.0	3217.2 ± 49.2
Cycle 2 (2018–2020)	2943.4 ± 118.3	2965.8 ± 75.3	2992.6 ± 167.0	5083.7 ± 596.8	4818.2 ± 308.6	5433.1 ± 230.6	3019.5 ± 130.2	3171.8 ± 118.7	3252.5 ± 76.5
Cycle 3 (2021–2023)	2907.5 ± 124.9 b	3234.6 ± 34.7 a	3203.2 ± 160.8 a	4924.9 ± 119.8 b	5005 ± 203.6 b	5904.1 ± 454.9 a	2612.7 ± 228.8 b	3238.8 ± 298.6 a	2660.8 ± 124.1 b
**Net income (USD ha^−2^)**									
Cycle1 (2015–2017)	1657.4 ± 84.1 a	1583.7 ± 51.3 a	1148.7 ± 75.6 b	3038 ± 117.8 a	2994.8 ± 630.1 a	2698.7 ± 67.5 a	1645.7 ± 29.8 a	1582.7 ± 67.0 a	1341.2 ± 49.2 b
Cycle 2 (2018–2020)	1563.3 ± 118.3 a	1480.7 ± 75.3 a	1077.1 ± 167.0 b	3638.2 ± 596.8 a	3267.8 ± 308.6 a	3557.2 ± 230.6 a	1574.1 ± 130.2 ab	1621.4 ± 118.7 a	1376.5 ± 76.5 b
Cycle 3 (2021–2023)	1527.4 ± 124.9 a	1749.5 ± 34.7 a	1287.6 ± 160.8 b	3479.5 ± 119.8 a	3454.5 ± 203.6 a	4028.1 ± 454.9 a	1167.2 ± 228.8 b	1688.3 ± 298.6 a	784.9 ± 124.1 b

Note: Values are means ± standard errors. The different lowercase letters indicate significant differences between the fertilization treatments at the *p* = 0.05 level. CK: chemical fertilizer alone; Straw: chemical fertilizer with returned straw; Straw + LGM: chemical fertilizer with returned straw and leguminous green manure; 1 USD = 7.21 CNY.

**Table 5:** The average nutrient contents in the LGM and straw of wheat, maize and sunflower (%).

	N	P	K
LGM	3.8 ± 0.08	0.35 ± 0.02	3.03 ± 0.06
Wheat	0.61 ± 0.02	0.15 ± 0.01	1.37 ± 0.04
Maize	0.65 ± 0.01	0.11 ± 0.01	1.62 ± 0.03
Sunflower	0.69 ± 0.01	0.11 ± 0.01	2.33 ± 0.05

Note: N: nitrogen; P: phosphorus; K: potassium; LGM: leguminous green manure.

## Data Availability

All data generated or used during the study appear in the submitted article.

## Supplementary Materials

The following supporting information can be downloaded at: https://www.mdpi.com/article/10.3390/plants13101358/s1, Table S1: Biomass of straw under different treatments and crop rotation cycles; Table S2: Biomass of green manure under different treatments and crop rotation cycles; Table S3: Inputs and outputs of wheat, maize, and sunflower with straw and green manure across the three cycles of wheat-maize-sunflower rotation system; Figure S1: Linear regression between crop yield and N use efficiency (a), P use efficiency (b) and K use efficiency (c), respectively. Bars represent standard errors. CK: chemical fertilizer alone; Straw: chemical fertilizer with returning straw; GM: chemical fertilizer with returning straw and green manure. N: nitrogen; P: phosphorus; K: potassium; Figure S2: Linear regression between crop yield and N harvest index (a), P harvest index (b) and K harvest index (c), respectively. Bars represent standard errors. CK: chemical fertilizer alone; Straw: chemical fertilizer with returning straw; GM: chemical fertilizer with returning straw and green manure. N: nitrogen; P: phosphorus; K: potassium.
