# Supplementary material for: Combined Application of Leguminous Green Manure and Straw Determined Grain Yield and Nutrient Use Efficiency in Wheat–Maize–Sunflower Rotations System in Northwest China
Source: Plants (Basel). 2024 May 14;13(10):1358. doi: 10.3390/plants13101358 (PMC11125438; doi:10.3390/plants13101358)
Supplement: Supplementary file 1 [file plants-13-01358-s001.zip › plants-3010723-supplementary.pdf]

Combined Application of Leguminous Green Manure and Straw Determined Grain Yield and Nutrient Use Efficiency in Wheat–Maize–Sunflower Rotations System in Northwest China

Table S1. Biomass of straw under different treatments and crop rotation cycles.

Rotation cycle	Treatment	Wheat (kg ha^{-1})	Maize (kg ha^{-1})	Sunflower (kg ha^{-1})
Cycle1	Straw	6444.3±269.4	19303.6±1042.6	9355.5±342.7
Cycle1	Straw + LGM	6380.9±181.4	22446.3±1569.2	9439.5±121.8
Cycle2	Straw	7053.3±149.5	18010.5±1023.4	9889.7±775.1
Cycle2	Straw + LGM	7243.9±95	21271.6±1002.8	10241.2±1273.3
Cycle3	Straw	6793.6±336.3	16135.8±2323.7	13587.2±748.5
Cycle3	Straw + LGM	6672.7±359.1	20942.1±1176	13561.6±883

Note: Values are means ± standard errors. Straw: chemical fertilizer with returning straw; Straw + LGM: chemical fertilizer with returning straw and green manure

Table S2. Biomass of green manure under different treatments and crop rotation cycles

Rotation cycle	Treatment	Wheat (kg ha^{-1})	Maize (kg ha^{-1})	Sunflower (kg ha^{-1})
cycle1	Straw + LGM	18065.14±2984.15	3765.83±966.52	9717.15±477.21
Cycle2	Straw + LGM	22783.64±917.7	3781.7±829.36	6375.32±487.2
Cycle3	Straw + LGM	24776.24±1233.2	880.25±36.5	7992.9±174.15

Note: Values are means ± standard errors. Straw + LGM: chemical fertilizer with returning straw and green manure

Table S3. Inputs and outputs of wheat, maize, and sunflower with straw and green manure across the three cycles of wheat-maize-sunflower rotation system.

Item	spring wheat			maize			sunflower			Price
	CK	Straw	Straw + LGM	CK	Straw	Straw + LGM	CK	Straw	Straw + LGM	
Average inputs										
Seed (kg ha^{-1})	375	375	375	45	45	45	30	30	30	\$ 0.7, 2.8, and 5.6 kg^{-1} for wheat, maize, and sunflower, respectively
Green manure seed (kg ha^{-1})	0	0	50	0	0	30	0	0	30	\$ 4.2 kg^{-1}
Urea (kg ha^{-1})	387	387	387	485	485	485	485	485	485	\$ 0.56 kg^{-1}
Diammonium phosphate (kg ha^{-1})	261	261	261	261	261	261	261	261	261	\$ 0.52 kg^{-1}
Potassium sulfate (kg ha^{-1})	180	180	180	180	180	180	180	180	180	\$ 0.46 kg^{-1}
Herbicide (bottle ha^{-1})	15	15	15	22.5	22.5	22.5	22.5	22.5	22.5	\$ 2.8 bottle^{-1}
Irrigation (m^3 ha^{-1})	1875	1875	1875	2250	2250	2250	1125	1125	1125	\$ 0.11 m^{-3}
Plastic film mulching (kg ha^{-1})	0	0	0	50	50	50	37.5	37.5	37.5	\$1.68 kg^{-1}
Labor (No. ha^{-1})	0	0	0	0	0	0	5	5	5	\$ 21 labor^{-1}
Machinery (times year^{-1})										

Rotary tillage	1	1	2	1	1	2	1	1	2	$ 94.5 ha^{-1}
Crushing	0	1	1	0	1	1	0	1	1	$ 105 ha^{-1}
Soil rolling	1	1	2	1	1	1	1	1	1	$ 21 ha^{-1}
Sowing	1	1	2	1	1	2	1	1	2	$105 ha^{-1}
Harvest	1	1	1	1	1	1	1	1	1	$105 ha^{-1}
Plough tillage	1	1	1	1	1	1	1	1	1	$105 ha^{-1}
Total ($ ha^{-2})	1380.1	1485.1	1915.6	1445.5	1550.5	1876	1445.5	1550.5	1876	
Average outputs, crop yield (Mg ha^{-1}, 13% ,14% of wheat, maize moisture content)										
Cycle1 (2015-2017)	6.78	6.85	6.84	15.25	15.46	15.56	3.68	3.73	3.83	$ 448, 294, and 840 Mg^{-1} for wheat, maize, and sunflower, respectively
Cycle 2 (2018-2020)	6.57	6.62	6.68	15.13	14.34	16.17	3.37	3.54	3.63	$ 448, 336, and 896 Mg^{-1} for wheat, maize, and sunflower, respectively
Cycle 3 (2021-2023)	6.49	7.22	7.15	13.53	13.75	16.22	2.17	2.69	2.21	$ 448, 364, and 1204 Mg^{-1} for wheat, maize, and sunflower, respectively
Net income ($ ha^{-2})										
Cycle1 (2015-2017)	1657.4±84.1a	1583.7±51.3a	1148.7±75.6b	3038±117.8a	2994.8±630.1a	2698.7±67.5a	1645.7±29.8a	1582.7±67.0a	1341.2±49.2b	
Cycle 2 (2018-2020)	1563.3±118.3a	1480.7±75.3a	1077.1±167.0b	3638.2±596.8a	3267.8±308.6a	3557.2±230.6a	1574.1±130.2ab	1621.4±118.7a	1376.5±76.5b	
Cycle 3 (2021-2023)	1527.4±124.9a	1749.5±34.7a	1287.6±160.8b	3479.5±119.8a	3454.5±203.6a	4028.1±454.9a	1167.2±228.8b	1688.3±298.6a	784.9±124.1b	

Note: Values are means ± standard errors. The different small letters indicate significant differences between different fertilization treatments at the P = 0.05 level. CK: chemical fertilizer alone; Straw: chemical fertilizer with returning straw; Straw + LGM: chemical fertilizer with returning straw and leguminous green manure; 1 USD $ = 7.21 CNY ¥.

Figure S1. Linear regression between crop yield and N use efficiency (a), P use efficiency (b) and K use efficiency (c), respectively. Bars represent standard errors. CK: chemical fertilizer alone; Straw: chemical fertilizer with returning straw; GM: chemical fertilizer with returning straw and green manure. N: nitrogen; P: phosphorus; K: potassium.

Figure S2. Linear regression between crop yield and N harvest index (a), P harvest index (b) and K harvest index (c), respectively. Bars represent standard errors. CK: chemical fertilizer alone; Straw: chemical fertilizer with returning straw; GM: chemical fertilizer with returning straw and green manure. N: nitrogen; P: phosphorus; K: potassium.

Figure S1

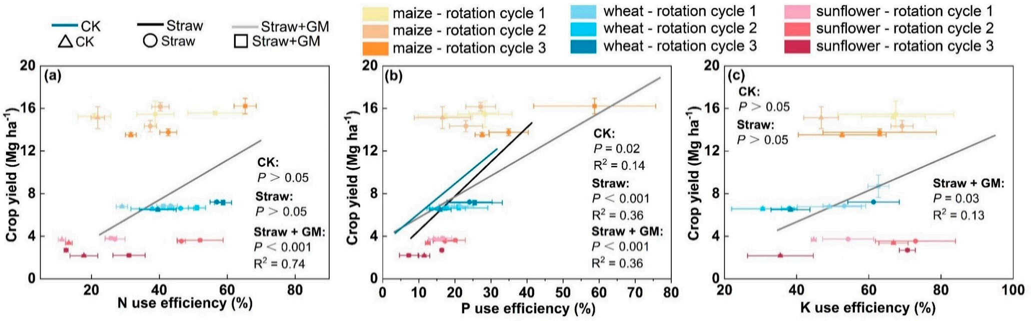

CK
Straw
Straw+GM
CK
Straw
Straw+GM
maize - rotation cycle 1
maize - rotation cycle 2
maize - rotation cycle 3
wheat - rotation cycle 1
wheat - rotation cycle 2
wheat - rotation cycle 3
sunflower - rotation cycle 1
sunflower - rotation cycle 2
sunflower - rotation cycle 3
(a)
Crop yield (Mg ha-1)
N use efficiency (%)
CK: P > 0.05
Straw: P > 0.05
Straw + GM: P < 0.001 R2 = 0.74
(b)
P use efficiency (%)
CK: P = 0.02 R2 = 0.14
Straw: P < 0.001 R2 = 0.36
Straw + GM: P < 0.001 R2 = 0.36
(c)
K use efficiency (%)
CK: P > 0.05
Straw: P > 0.05
Straw + GM: P = 0.03 R2 = 0.13

Figure S2

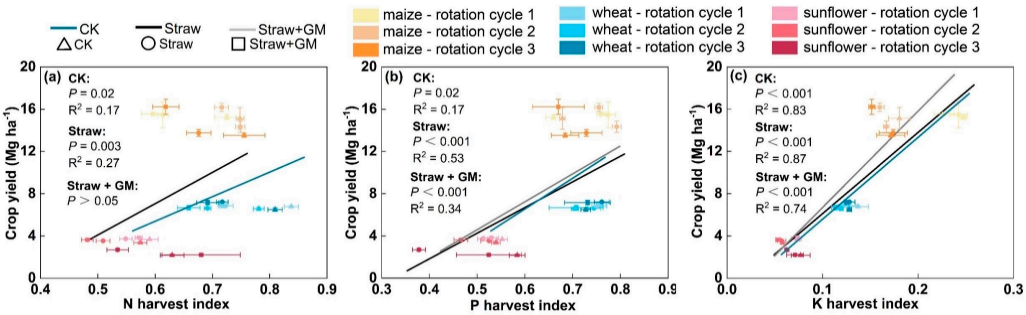

CK
Straw
Straw+GM
CK
Straw
Straw+GM
maize - rotation cycle 1
maize - rotation cycle 2
maize - rotation cycle 3
wheat - rotation cycle 1
wheat - rotation cycle 2
wheat - rotation cycle 3
sunflower - rotation cycle 1
sunflower - rotation cycle 2
sunflower - rotation cycle 3
(a)
CK:
P = 0.02
R2 = 0.17
Straw:
P = 0.003
R2 = 0.27
Straw + GM:
P > 0.05
Crop yield (Mg ha-1)
N harvest index
(b)
CK:
P = 0.02
R2 = 0.17
Straw:
P < 0.001
R2 = 0.53
Straw + GM:
P < 0.001
R2 = 0.34
P harvest index
(c)
CK:
P < 0.001
R2 = 0.83
Straw:
P < 0.001
R2 = 0.87
Straw + GM:
P < 0.001
R2 = 0.74
K harvest index
